# Trends in survival after heart transplantation based on Social Vulnerability Index in the United States

**DOI:** 10.1016/j.jhlto.2024.100079

**Published:** 2024-03-02

**Authors:** Lovette Azap, Adrian Diaz, Doug A. Gouchoe, Nahush A. Mokadam, Sakima Smith, Matthew C. Henn, Bryan A. Whitson, Alim Habib, Brent C. Lampert, Timothy M. Pawlik, Asvin M. Ganapathi

**Affiliations:** a Division of Cardiac Surgery, Department of Surgery, The Ohio State University Wexner Medical Center, Columbus, OH; b Division of Cardiovascular Medicine, Department of Medicine, The Ohio State University Wexner Medical Center, Columbus, OH; c Division of Surgical Oncology, Department of Surgery, The Ohio State University Wexner Medical Center and James Cancer Center, Columbus, OH

**Keywords:** cardiac, transplant, surgical outcomes, Social Vulnerability Index, Social determinants of health

## Abstract

**Background:**

The association of social vulnerability (SV) and cardiac transplant survival remains poorly defined, particularly related to long-term outcomes. The purpose of this study was to define the impact of SV on survival among heart transplant recipients with at least 1 year of survival post-transplant.

**Methods:**

Heart transplant recipients were identified using the United Network for Organ Sharing database between June 1, 2006, and December 31, 2020. The Center for Disease Control’s Social Vulnerability Index (SVI) database was used to stratify patients based on SVI into 3 groups: low: <25; average: 26 to 74; high: 75+. The groups were analyzed with comparative statistics, and unadjusted survival was assessed using Kaplan-Meier methods. To determine the independent association between SVI and survival, a multivariable Cox proportional hazard model was created.

**Results:**

There were 27,740 recipients identified. High SVI patients more commonly identified as Black individuals and had a higher incidence of diabetes, pretransplant intensive care unit admission, and need for concomitant kidney transplant (*p* < 0.05 for all). Additionally, high SVI patients had the longest length of stay post-transplant (21.4 days) (*p* < 0.05). High and average SVI patients had inferior 3-year, 5-year, and 10-year survival vs low SVI patients (*p* < 0.05). After adjustment, average (hazard ratio [HR]: 1.12) and high (HR: 1.16) SVI were independently associated with an increased risk of mortality on multivariable analysis (both *p* < 0.001).

**Conclusion:**

High or average SVI is independently associated with increased mortality following heart transplantation in patients with 1-year conditional survival. These findings demonstrate that disparities persist among heart transplant recipients during long-term follow-up.

## Background

Heart transplantation is a life-saving therapy for patients with end-stage heart failure. Despite surgical advancements within thoracic transplantation surgery, inequities in access to surgical care and disparities in patient health outcomes persist among vulnerable patient populations.^1^, ^2^ Social determinants of health (SDOH) have been identified as critical contributors to disparities in surgical outcomes.^3^ Specifically, patient-level factors, such as socioeconomic status, insurance status, geographical location, housing, and education, have been associated with worse access to optimal health care among vulnerable patient populations.^4^, ^5^, ^6^, ^7^

In 2011, the US Centers for Disease Control and Prevention (CDC) developed a novel metric, the Social Vulnerability Index (SVI). SVI is comprised of 16 U.S. census components, which are categorized into 4 overarching subthemes of socioeconomic status, household composition and disability, minority status and language, as well as housing type and transportation.^8^ Ultimately, by utilizing various socioeconomic and environmental factors, SVI measures residential-level vulnerability. Though initially created for emergency response usage, SVI has been demonstrated to have utility in evaluating the impact of social and environmental deprivation on patient-level health outcomes across an array of surgical specialties, such as surgical oncology and colorectal surgery.^9^, ^10^ Previous studies have evaluated SVI relative to patient health outcomes and have demonstrated that SVI may serve as an accurate measure of SDOH given how it incorporates several important factors relative to SDOH.^9^, ^10^ A recent regional study by Shin et al in 2024 found that increased SVI is associated with disparities in access to surgical care.^11^ Another regional study from Carmichael et al in 2020 demonstrated that residents of high social vulnerability regions have a greater likelihood of undergoing nonelective surgical operations and worse postoperative surgical outcomes.^12^ Moreover, neighborhood socioeconomic disparities have also resulted in disparities with management of treatment long-term due to a greater risk of medication noncompliance because of financial constraints,^13^ which can be incredibly impactful in transplant recipients.

Patients who reside in areas of greater SVI may experience an increased risk of postoperative complications, long-term mortality, and emergent admission for surgery.^9^, ^10^, ^11^, ^12^, ^13^ However, the role of social vulnerability among heart transplant recipients remains ill-defined. Given that recipients of heart transplantation have a median survival approaching 13 years, they require complex multidisciplinary care and monitoring for the duration of their life following transplantation and social vulnerability may be an important driver of post-transplant outcomes, even for patients surviving at least 1 year. Thus, the objective of this study was to determine the association of social vulnerability (at the time of transplant) with long-term outcomes among recipients with at least 1 year of survival following heart transplantation. We hypothesized that patients residing in areas of greater SVI would have inferior survival and outcomes when compared to patients from low SVI regions.

## Material and methods

### Data source

The United Network for Organ Sharing (UNOS)/Organ Procurement and Transplantation Network (OPTN) database was utilized to acquire donor, recipient, and transplant information. Recipient zip codes within the UNOS database were associated with the corresponding SVI with the aid of the CDC’s SVI database. SVI is a composite measure of vulnerability and is on a scale of 1 to 100, where a greater value indicates increased social vulnerability. The institutional review board at The Ohio State University Wexner Medical Center deemed this study as exempt (IRB#2018H0079).

### Study population

Adult heart transplant recipients with at least 1 year of survival following transplantation from June 1, 2006, to December 31, 2021, were identified from the UNOS/OPTN database. Heart transplant recipients with age <18, redo transplants, or concomitant lung or liver transplants were excluded from this analysis. The UNOS/OPTN database was merged with the CDC’s SVI database to obtain clinical and geographical information on each patient’s county of residence at the time of heart transplantation. Patients were stratified into 3 cohorts relative to SVI of residence prior to transplantation: low (25 or less), average (26-74), and high (greater than 75), based on CDC classifications and previous work with SVI.^9^, ^10^

### Outcomes

The primary outcome of interest was 3-, 5-, and 10-year survival following transplantation, and its subsequent association with SVI. Survival was calculated using transplant data and follow-up data provided in the UNOS/OPTN dataset where patients are classified as alive, dead, or lost to follow-up. Secondary outcomes of interest included perioperative characteristics, such as ischemic time, length of stay, postoperative dialysis, postoperative stroke, postoperative pacemaker, acute rejection, and treatment for rejection within the first year.

## Calculation

Continuous and categorical variables were detailed as mean or median (based on normality) and frequency, respectively. Comparative statistics were used to analyze differences among the groups relative to recipient, donor, and transplant characteristics. To assess survival, Kaplan-Meier methods with the log-rank test were employed for unadjusted analysis. To adjust for other covariates, a multivariable Cox proportional hazard model was developed to evaluate the independent association between SVI and longitudinal survival. Variables included in the model were chosen a priori based on prior work and clinical expertise. These variables included SVI status, recipient/donor age, recipient/donor gender, race, insurance status, body mass index (BMI), diabetes, glomerular filtration rate (GFR), panel reactive antibodies (PRA), functional status, mechanical circulatory support, smoking history, ischemic time, acute rejection within the first year, and era of transplantation. Median SVI by recipient zip code was plotted by state to determine if any regional trends were apparent. All analyses were performed in Stata 16 (College Station, TX). All tests were 2-sided and significance was determined by a value of *p* < 0.05.

## Results

### Recipient and donor baseline characteristics

**Figure 1:**
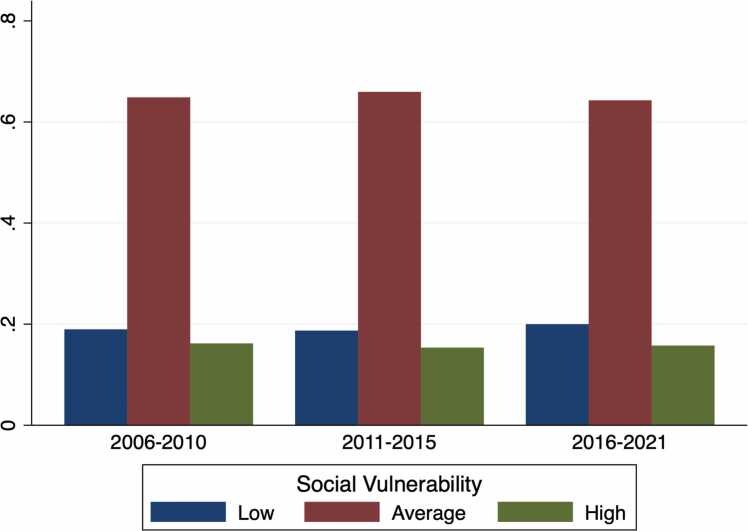
Bar graph by era of distribution of Social Vulnerability Index (SVI).

**Figure 2:**
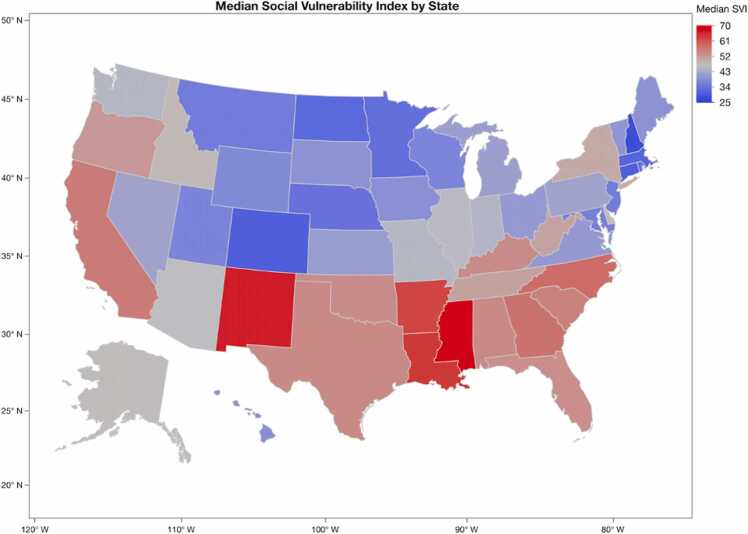
Geographic distribution of recipient Social Vulnerability Index (SVI) by state.

There were 27,470 heart transplant recipients who met the criteria for inclusion during the study period. Overall, 5,354 (19.5%) patients resided in low SVI areas, 18,024 (65.6%) in average SVI areas, and 4,362 (15.9%) in high SVI areas. The percentage of patients in each SVI strata was relatively similar among all eras (Figure 1). Examination of median SVI by recipient state demonstrated that southern states (Mississippi, Louisiana, and Arkansas) had the highest median SVI for heart transplantation recipients during the study period (Figure 2).

Recipients with high SVI were younger, less commonly male, and more often identified as Black individuals (*p* < 0.001 for all). Additionally, high SVI recipients more often had public insurances, such as Medicare or Medicaid (*p* < 0.001 for both). Comorbid disease incidence, including history of smoking (45.8%; *p* < 0.001), diabetes (30.9%; *p* < 0.001), and preoperative dialysis (4.1%; *p* < 0.001), were higher in the high SVI cohort. Additionally, high SVI recipients were more often admitted to the intensive care unit prior to transplant (*p* = 0.002) (Table 1). With regards to donor characteristics, there were no significant differences in age, sex, history of hypertension, or recent cocaine use (all *p* > 0.05). Donors for recipients residing within low SVI regions had significantly higher incidence of a smoking history (>20 pack years) when compared to donors from average or high SVI regions (low: 13.5%; average: 13.0%; high: 11.7%) (Table 2).

**Table 1:** Recipient Demographics and Baseline Characteristics

Variables	All patients (N = 27,740)	Low SVI N = 5,354	Average SVI (N = 18,024)	High SVI (N = 4,362)	*p*-value
Age (SD)	56.0 (46.0-63.0)	58.0 (49.0-64.0)	56.0 (46.0-63.0)	54.0 (44.0-61.0)	<0.001
Male (*n*, %)	20,616 (74.3%)	4,094 (76.5%)	13,406 (74.4%)	3,116 (71.4%)	<0.001
Race (*n*, %)					
White	18,260 (65.8%)	4,536 (84.7%)	12,360 (68.6%)	1,364 (31.3%)	<0.001
Black	6,023 (21.7%)	453 (8.5%)	3,636 (20.2%)	1,934 (44.3%)	
Other	3,457 (12.5%)	365 (6.8%)	2,028 (11.3%)	1,064 (24.4%)	
Insurance status (*n*, %)					
Private	13,744 (49.5%)	3,266 (61.0%)	8,914 (49.5%)	1,564 (35.9%)	<0.001
Medicare	9,541 (34.4%)	1,620 (30.3%)	6,289 (34.9%)	1,632 (37.4%)	
Medicaid	3,480 (12.5%)	336 (6.3%)	2,150 (11.9%)	994 (22.8%)	
Other	968 (3.5%)	132 (2.5%)	666 (3.7%)	170 (3.9%)	
Smoking history (*n*, %)	12,814 (46.2%)	2,364 (44.3%)	8,458 (47.1%)	1,992 (45.8%)	0.001
Diabetes (*n*, %)	7,741 (27.9%)	1,332 (24.9%)	5,062 (28.1%)	1,347 (30.9%)	<0.001
BMI (SD)	27.4 (4.9)	27.1 (4.7)	27.4 (4.9)	27.4 (5.0)	<0.001
GFR (SD)	66.6 (33.0)	65.8 (31.9)	66.7 (33.1)	67.0 (34.4)	0.15
Preoperative dialysis (*n*, %)	907 (3.3%)	137 (2.6%)	591 (3.3%)	179 (4.1%)	<0.001
Hospitalization (*n*, %)					
Not hospitalized	14,468 (52.2%)	2,779 (51.9%)	9,510 (52.8%)	2,179 (50.0%)	0.002
Hospitalized	4,297 (15.5%)	881 (16.5%)	2,729 (15.1%)	687 (15.7%)	
ICU	8,975 (32.4%)	1,694 (31.6%)	5,785 (32.1%)	1,496 (34.3%)	
Diagnosis (*n*, %)					
Ischemic	9,707 (35.0%)	1,955 (36.5%)	6,429 (35.7%)	1,323 (30.3%)	<0.001
Nonischemic	14,454 (52.1%)	2,476 (46.2%)	9,359 (51.9%)	2,619 (60.0%)	
Congenital	32 (0.1%)	9 (0.2%)	19 (0.1%)	4 (0.1%)	
Other	3,547 (12.8%)	914 (17.1%)	2,217 (12.3%)	416 (9.5%)	
Waitlist time (SD)	214.6 (360.4)	223.5 (367.6)	214.3 (361.5)	205.1 (346.3)	0.043
Combined PRA (SD)	8.5 (20.5)	7.6 (19.7)	8.4 (20.3)	9.8 (22.1)	<0.001

Abbreviations: BMI, body mass index; GFR, glomerular filtration rate; ICU, intensive care unit; PRA*, panel-reactive antibody; SD, standard deviation; SVI, Social Vulnerability Index.*

Categorical variables are displayed as number (%). Continuous variables are displayed as median (25th percentile, 75th percentile).

**Table 2:** Donor Characteristics

Characteristics	All patients (N = 27,740)	Low SVI N = 5,354	Average SVI (N = 18,024)	High SVI (N = 4,362)	*p*-value
Age (SD)	31.8 (11.3)	32.1 (11.3)	31.8 (11.3)	31.7 (11.1)	0.18
Male gender (*n*, %)	19,823 (71.5%)	3,825 (71.4%)	12,909 (71.6%)	3,089 (70.8%)	0.57
Hypertension (*n*, %)	4,133 (14.9%)	765 (14.3%)	2,694 (14.9%)	674 (15.5%)	0.52
Cocaine use (*n*, %)	5,425 (19.6%)	1,072 (20.0%)	3,486 (19.3%)	867 (19.9%)	0.11
Smoking history (>20 PY) (*n*, %)	3,567 (12.9%)	722 (13.5%)	2,335 (13.0%)	510 (11.7%)	0.03

Abbreviations: PY, Pack Years; SD, standard deviation; SVI, Social Vulnerability Index.

Categorical variables are displayed as number (%). Continuous variables are displayed as median (25th percentile, 75th percentile).

### Peri-operative outcomes

Regarding transplant characteristics, those in the high SVI group had statistically significantly longer ischemic times (low: 3.1 hours; average: 3.2 hours; high: 3.2 hours) (*p* = 0.004). Heart transplant recipients in the high SVI group had an increased incidence of concomitant kidney transplant as compared to recipients in low SVI areas (3.5%) (*p* < 0.001). Additionally, high SVI patients experienced a greater length of stay (21.4 days) when compared to patients residing in average (20.3 days) or low SVI regions (20 days) (*p* = 0.004). No significant differences were noted in incidence of postoperative dialysis, stroke, or pacemaker placement. While the incidence of rejection during index hospitalization was significantly higher in the average (19.1%) and high (19.1%) SVI groups (*p* = 0.02), the incidence of treatment for rejection within the first year was not significantly different (*p* = 0.05) (Table 3).

**Table 3:** Peri-Operative Characteristics

Variables	All patients (N = 27,740)	Low SVI N = 5,354	Average SVI (N = 18,024)	High SVI (N = 4,362)	*p*-value
Concomitant kidney transplant (n, %)	1,202 (4.3%)	190 (3.5%)	787 (4.4%)	225 (5.2%)	<0.001
Transplant center SVI (n, %)	50.9 (24.7)	52.2 (24.7)	50.0 (24.1)	52.8 (26.9)	<0.001
Ischemic time	3.2 (1.0)	3.1 (1.0)	3.2 (1.0)	3.2 (1.1)	0.004
Length of stay	20.4 (22.2)	20.0 (22.9)	20.3 (21.6)	21.4 (23.6)	0.004
Distance	186.1 (213.7)	182.3 (216.0)	187.0 (213.3)	187.3 (212.5)	0.34
Postoperative dialysis	2,333 (8.4%)	413 (7.7%)	1,552 (8.6%)	368 (8.4%)	0.36
Postoperative stroke	548 (2.0%)	90 (1.7%)	370 (2.1%)	88 (2.0%)	0.38
Postoperative pacemaker	805 (2.9%)	170 (3.2%)	524 (2.9%)	111 (2.5%)	0.15
Acute rejection during hospitalization	5,190 (18.7%)	916 (17.1%)	3,442 (19.1%)	832 (19.1%)	0.02
Treated rejection in first year	5,102 (18.4%)	914 (17.1%)	3,376 (18.7%)	812 (18.6%)	0.05

Abbreviations: SVI, Social Vulnerability Index.

Categorical variables are displayed as number (%). Continuous variables are displayed as median (25th percentile, 75th percentile).

### Unadjusted long-term survival

**Figure 3:**
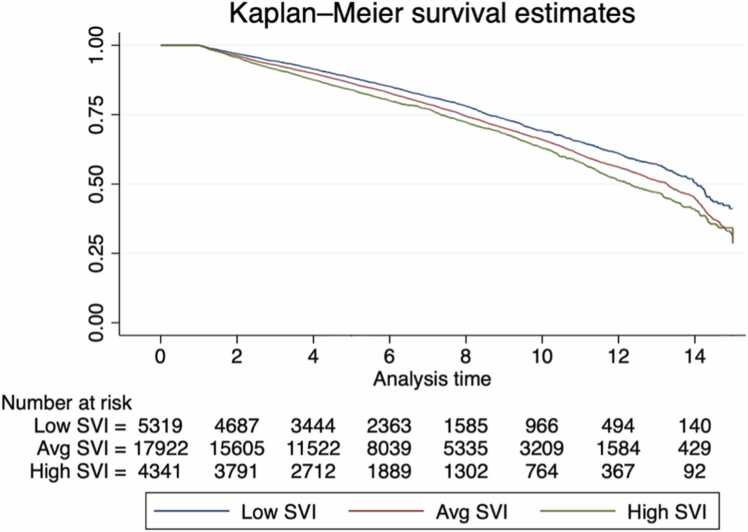
Unadjusted Kaplan-Meier curves depicting 3-, 5-, and 10-year survival estimates by Social Vulnerability Index (SVI) level: low, average, and high.

Unadjusted survival analysis revealed that at 3, 5, and 10 years, those in the high SVI cohort had significantly lower survival when compared to the other groups (*p* < 0.001, Figure 3). At 5 years, survival was 88.4% (95% confidence interval (CI): 87.4%-89.4%) for the low SVI group, 86.4% (95% CI: 85.9%-87.05) for the average SVI group, and 84.0% (95% CI: 82.3%-85.2%) for the high SVI group. At 10 years, survival was 69.7% (95% CI: 67.8%-71.5%) for the low SVI group, 66.4% (95% CI: 65.4%-67.4%) for the average SVI group, and 63.3% (95% CI: 61.2%-65.3%) for the high SVI group.

### Adjusted analysis

Following adjustment, Cox regression modeling demonstrated that residence in average (hazard ratio [HR]: 1.12, 95% CI: 1.05-1.20, *p* = 0.001) and high (HR: 1.16, 95% CI: 1.06-1.27, *p* = 0.001) SVI areas were independently associated with an increased risk of mortality when compared to patients who resided in low SVI areas. Older (HR: 1.005, 95% CI: 1.003-1.008, *p* < 0.001) and male (HR: 1.093, 95% CI: 1.022-1.169, *p* = 0.010) patients also had a higher mortality risk vs patients who were younger and female. Furthermore, heart transplant recipients who identified as Black race were at an increased risk of mortality compared with Caucasian patients (HR: 1.206, 95% CI: 1.130-1.286, *p* < 0.001). In contrast, heart transplant recipients who identified as other have a decreased risk of mortality compared with Caucasian patients (HR: 0.854, 95% CI: 0.784-0.930, *p* < 0.001). Patients with Medicare (HR: 1.285, 95% CI: 1.215-1.360), Medicaid (HR: 1.472, 95% CI: 1.361-1.592), and other forms of insurance (HR: 1.160, 95% CI: 1.009-1.334) were at greater risk of mortality vs patients with private insurance following heart transplantation (all *p* < 0.05). Recipients with a greater BMI (HR: 1.009, 95% CI: 1.003-1.014), a diagnosis of diabetes prior to transplant (HR: 1.379, 95% CI: 1.306-1.457), and increased PRA (HR: 1.002, 95% CI: 1.000-1.003) also demonstrated an association with a greater mortality risk (all *p* < 0.05). Patients who received grafts from donors who were older (HR: 1.009, 95% CI: 1.007-1.011) and had longer ischemic times (HR: 1.034, 95% CI: 1.010-1.059) experienced a greater risk of mortality as well. In contrast, recipients of grafts from female donors experienced a decreased risk of mortality (HR: 0.937, 95% CI: 0.881-0.997, *p* = 0.039). In addition, patients who received a transplant within the 2016 to 2021 era (HR: 1.238, 95% CI: 1.136-1.349) were at a greater risk of mortality when compared with patients who had transplants between 2006 and 2020 (*p* < 0.001) (Table 4).

**Table 4:** Cox Regression Model Output

Variable	Reference	Hazard ratio	Lower 95% CI	Upper 95% CI	*p*-value
Recipient SVI, average	SVI, Low	1.119	1.045	1.200	0.001
Recipient SVI, high		1.159	1.059	1.268	0.001
Recipient age (per 10 years)		1.005	1.003	1.008	<0.001
Recipient gender, male	Female	1.093	1.022	1.169	0.010
Recipient race, Black	Caucasian	1.206	1.130	1.286	<0.001
Recipient race, other	Caucasian	0.854	0.784	0.930	<0.001
Recipient insurance, Medicare	Private	1.285	1.215	1.360	<0.001
Recipient insurance, Medicaid	Private	1.472	1.361	1.592	<0.001
Recipient insurance, other	Private	1.160	1.009	1.334	0.038
Recipient BMI		1.009	1.003	1.014	0.002
Recipient diabetes		1.379	1.306	1.456	<0.001
Recipient GFR at transplant		0.999	0.998	1.000	0.188
Recipient PRA at transplant		1.002	1.000	1.003	0.014
Recipient functional status, disabled/hospitalized	Not hospitalized	1.061	0.999	1.128	0.054
Recipient acute rejection	No acute rejection	0.816	0.726	0.918	0.001
Donor age (per 10 years)		1.009	1.007	1.011	<0.001
Donor gender, female	Male	0.937	0.881	0.997	0.039
Donor history of smoking		1.060	0.988	1.139	0.106
Donor graft ischemic time (per 1 hour)		1.034	1.010	1.059	0.006
Transplant era, 2011-2015	Era, 2006-2010	1.022	0.961	1.087	0.487
Transplant era, 2016-2021	Era, 2006-2010	1.238	1.136	1.349	<0.001

Abbreviations: BMI, body mass index; CI, confidence interval; GFR, glomerular filtration rate; PRA*,* panel-reactive antibody; SVI, Social Vulnerability Index.

## Discussion

Disparities in access to care, socioeconomic status, and geography persist and contribute toward health care inequities among vulnerable patient populations.^4^, ^5^, ^6^, ^7^ SDOH define conditions where communities work, learn, and live and have been implicated in disparate health outcomes across numerous surgical specialties.^14^, ^15^ Patients who reside in geographical locations of greater social vulnerability face adverse SDOH that serve as barriers to accessing health care resources, such as inadequate transportation, low-income, and lack of access to appropriate preventive care opportunities.^14^, ^15^ This lack of access can be especially important among transplant recipients as these patients require follow-up for the duration of their life to monitor for rejection, graft function, and infection.

Notably, the findings within this study demonstrate that greater exposure to social vulnerability was independently associated with a long-term mortality among heart transplant recipients residing in average and high social vulnerability regions when compared to recipients within low social vulnerability regions. The unadjusted survival analysis also demonstrated that median survival was extended by 1 year for each of the groups, as the level of SVI decreased. Although increased SVI was independently associated with increased mortality, it should be noted that race was also independently associated with mortality with Black recipients having increased mortality hazard. Furthermore, patients who had forms of federal health insurance, such as Medicare and Medicaid, also had increased hazard of mortality following heart transplantation when compared to recipients who had private health insurance. Prior studies have demonstrated that the highest risk of mortality occurs within the first year following heart transplantation, which is typically caused by graft function.^16^ However, as this cohort was comprised of recipients who survived at least 1 year following transplantation, these results indicate that factors outside of solely graft function, such as SDOH, may have driven disparate outcomes across the strata of SVI.

The relative health of communities located within regions of greater exposure to social vulnerability may also drive disparities associated with perioperative outcomes. Existing literature has reported that high SVI regions are associated with increased risk factors for cardiovascular disease, such as diabetes, poor exercise, and smoking. This finding is consistent with the present study as our findings demonstrated that high SVI heart transplant recipients presented at a younger age with a greater comorbid disease burden. Prior studies have noted that patients with a higher incidence of comorbid conditions are predisposed to suboptimal surgical outcomes, including textbook outcomes and postoperative complications.^9^, ^17^ These findings may be exacerbated by a disproportionately lower ability for impoverished patients to access preventative treatment and manage chronic diseases, both pre- and post-transplant which are critical in preventing the progression of cardiovascular disease and associated comorbidities.^18^ Patients with pre-existing diabetes commonly see their diabetes worsen due to chronic steroid usage and if not appropriately controlled this may lead to worsening diabetes and impact other outcomes.

Individual-level variants in insurance status may affect health care utilization by patients, contributing toward disparities in overall survival. Prior studies have demonstrated that patients with public insurance, such as Medicare and Medicaid, are more likely to seek health care services at emergency departments and less likely to visit outpatient settings.^19^ This pattern of health care utilization indicates that Medicare and Medicaid beneficiaries may face additional barriers to accessing outpatient care services, which would ultimately enable adequate long-term management of chronic diseases, like post-transplant care.^19^ Furthermore, patients who visit emergency rooms for care will commonly have a higher level of acuity than those seen in outpatient clinics and for transplant recipients could represent later presentation of acute issues. Data from this analysis demonstrated that Medicare and Medicaid patients experienced greater risk of long-term mortality following heart transplantation compared with patients with private insurance, which may corroborate previous findings. While all recipients undergo education regarding the importance of follow-up within transplant clinics, a better understanding of the social vulnerability of a patient may help guide transplant centers and coordinators to focus on increased resources to those most vulnerable. Such resources may be in the form of transportation assistance or more frequent check-ins. Furthermore, these disparities may also impact medication adherence, which in transplantation can manifest with immunosuppressive therapies.^19^, ^20^ Although potential recipients are screened for financial viability prior to transplant, the possibility of recipients in high SVI areas facing a greater burden of accessing medications due to financial strain remains a possibility.^20^ Medication adherence is not limited to immunosuppression only. Heart transplant recipients are susceptible to chronic rejection through coronary disease as well as other comorbidities.^21^, ^22^ Thus, adherence or lack thereof to important medications, such as statins, may also impact survival among this patient cohort.

Although recipients who identify as Black have the highest rates of heart failure, they have the lowest rates of receiving heart transplantation.^23^ In addition to the findings regarding SVI, our analysis demonstrated increased mortality hazard for Black recipients with 1-year conditional survival. As numerous mechanisms have been proposed, such as SDOH, genetic mismatch, access to care, and donor and recipient characteristics, UNOS developed revisions to the allocation system which expanded access to organs for the most medically urgent patients and mitigate disparities.^24^, ^25^, ^26^, ^27^ However, Chouairi et al conducted a national study using the UNOS database that demonstrated that although a greater number of minority patients were listed for transplantation, disparities persisted as recipients who identified as Black were still less likely to be transplanted and had greater risk of mortality following transplantation.^23^ These findings, as well as the findings within our own analysis, highlight the need for a better understanding of the interaction between race and social vulnerability.

Ultimately, as providers the best way to affect change is to understand the causes behind disparities and issues. To date, there is a paucity of literature on what limitations socially vulnerable recipients face. As a transplant community, it would be of great utility to better understand the limitations our recipients face, particularly when it comes to areas of known disparities, such as social vulnerability or race. Prior literature has emphasized the importance of increasing minority involvement within clinical trials, improving social policies that may drive health disparities, and improving the number of diverse health care professionals within the medical field.^23^, ^24^, ^28^ Furthermore for transplant centers that serve more socially vulnerable patients, increased resources for coordinators, case managers, and social workers may help to positively impact these patients.

### Study limitations

It is important to acknowledge several limitations that could impact the results of the current study. As with any study that uses administrative databases, these findings are subject to residual confounding bias due to unmeasured factors, such as noncoded comorbidities and complications. Furthermore, patient-level SVI was determined utilizing their zip code at the time of transplantation which has several limitations. While there likely is variation in SVI over time analysis of SVI change over time demonstrated that the median absolute change was 6.7% from 2010 to 2020 with an IQR of 2.9% to 12.9%. Secondarily, due to limitations within the UNOS database, we cannot account for recipients who moved during the follow-up period, that is, from a county of low to high SVI and vice-versa, which could possibly affect our results. Additionally, the choice of 1 year for conditional survival was based on prior analyses, however conditional survival based on a shorter (6 months) or longer (2 years) time frame could impact the findings. Lastly, SVI does not capture the actual socioeconomic status of the individual recipient and therefore more studies using single institutional databases (which contain granular data) are needed to clarify the association of SVI and heart transplantation outcomes. Finally, our study did not evaluate follow-up visits, rehospitalization, or compliance to immunosuppression, which may impact the outcomes.

## Conclusions

Data from the current study demonstrate that as SVI-level increased, the risk of 3-, 5-, and 10-year mortality increased, even following adjustment for other covariates. Given this difference in long-term survival, even in patients who survive at least 1-year post-transplant, further research is necessary to better understand the driving mechanisms behind these disparities.

## Disclosure statement

The authors declare the following financial interests/personal relationships which may be considered as potential competing interests: Asvin Ganapathi reports financial support was provided by Health Resources and Services Administration. Asvin Ganapathi reports a relationship with AbbVie Inc that includes consulting or advisory. Bryan A. Whitson reports a relationship with TransMedics Inc that includes consulting or advisory. Nahush A. Mokadam reports a relationship with Abbott that includes consulting or advisory. Nahush A. Mokadam reports a relationship with Medtronic that includes consulting or advisory. Nahush A. Mokadam reports a relationship with CARMAT that includes consulting or advisory. Nahush A. Mokadam reports a relationship with XyloCor Therapeutics Inc that includes consulting or advisory. Nahush A. Mokadam reports a relationship with SynCardia Systems LLC that includes consulting or advisory. The other authors declare that they have no known competing financial interests or personal relationships that could have appeared to influence the work reported in this paper.

The authors thank Jeffrey Sneddon for helping obtain the UNOS/OPTN STAR file used for analysis. The content is the responsibility of the authors alone and does not necessarily reflect the views or policies of the Department of Health and Human Services, nor does mention of trade names, commercial products or organizations imply endorsement by the U.S. Government.

This work was supported in part by the 10.13039/100000102Health Resources and Services Administration contract HHSH250-2019-00001C.
